# Supplementary material for: A fault-intrusion-tolerant system and deadline-aware algorithm for scheduling scientific workflow in the cloud
Source: PeerJ Comput Sci. 2021 Nov 2;7:e747. doi: 10.7717/peerj-cs.747 (PMC8576554; doi:10.7717/peerj-cs.747)
Supplement: Supplemental Information 6 [file peerj-cs-07-747-s006.zip › Myproject1.1/docs/org/cloudbus/cloudsim/power/package-tree.html]

org.cloudbus.cloudsim.power Class Hierarchy


JavaScript is disabled on your browser.


- Overview
- Package
- Class
- Use
- Tree
- Deprecated
- Index
- Help

- Prev
- Next

- Frames
- No Frames

- All Classes

# Hierarchy For Package org.cloudbus.cloudsim.power

Package Hierarchies:

- All Packages

## Class Hierarchy

- java.lang.Object
  - org.cloudbus.cloudsim.Host
    - org.cloudbus.cloudsim.HostDynamicWorkload
      - org.cloudbus.cloudsim.power.PowerHost
        - org.cloudbus.cloudsim.power.PowerHostUtilizationHistory
  - org.cloudbus.cloudsim.power.PowerVmSelectionPolicy
    - org.cloudbus.cloudsim.power.PowerVmSelectionPolicyMaximumCorrelation
    - org.cloudbus.cloudsim.power.PowerVmSelectionPolicyMinimumMigrationTime
    - org.cloudbus.cloudsim.power.PowerVmSelectionPolicyMinimumUtilization
    - org.cloudbus.cloudsim.power.PowerVmSelectionPolicyRandomSelection
  - org.cloudbus.cloudsim.core.SimEntity (implements java.lang.Cloneable)
    - org.cloudbus.cloudsim.Datacenter
      - org.cloudbus.cloudsim.power.PowerDatacenter
        - org.cloudbus.cloudsim.power.PowerDatacenterNonPowerAware
    - org.cloudbus.cloudsim.DatacenterBroker
      - org.cloudbus.cloudsim.power.PowerDatacenterBroker
  - org.cloudbus.cloudsim.Vm
    - org.cloudbus.cloudsim.power.PowerVm
  - org.cloudbus.cloudsim.VmAllocationPolicy
    - org.cloudbus.cloudsim.power.PowerVmAllocationPolicyAbstract
      - org.cloudbus.cloudsim.power.PowerVmAllocationPolicyMigrationAbstract
        - org.cloudbus.cloudsim.power.PowerVmAllocationPolicyMigrationInterQuartileRange
        - org.cloudbus.cloudsim.power.PowerVmAllocationPolicyMigrationLocalRegression
          - org.cloudbus.cloudsim.power.PowerVmAllocationPolicyMigrationLocalRegressionRobust
        - org.cloudbus.cloudsim.power.PowerVmAllocationPolicyMigrationMedianAbsoluteDeviation
        - org.cloudbus.cloudsim.power.PowerVmAllocationPolicyMigrationStaticThreshold
      - org.cloudbus.cloudsim.power.PowerVmAllocationPolicySimple

- Overview
- Package
- Class
- Use
- Tree
- Deprecated
- Index
- Help

- Prev
- Next

- Frames
- No Frames

- All Classes
